# Daily Exposure to Dust Alters Innate Immunity

**DOI:** 10.1371/journal.pone.0031646

**Published:** 2012-02-15

**Authors:** Karin Sahlander, Kjell Larsson, Lena Palmberg

**Affiliations:** Lung and Allergy Research, National Institute of Environmental Medicine, Karolinska Institutet, Stockholm, Sweden; Charité-University Medicine Berlin, Germany

## Abstract

Pig farmers are exposed to organic material in pig barns on a daily basis and have signs of an ongoing chronic airway inflammation and increased prevalence of chronic inflammatory airway diseases, predominantly chronic bronchitis. Interestingly, the inflammatory response to acute exposure to organic dust is attenuated in farmers. The aim of the study was to closer characterize innate immunity features in blood and airways in farmers and in naïve, non-exposed, controls. The expression of pattern recognition receptors (TLR2, TLR4 and CD14) whose ligands are abundant in pig barn dust and adhesion proteins (CD11b, CD62L and CD162L) on blood and sputum neutrophils in pig farmers and soluble TLR2 and CD14 (sTLR2 and sCD14) in blood and sputum were assessed in pig farmers and previously unexposed controls. The release of pro-inflammatory cytokines from blood cells stimulated with LPS *ex vivo* was measured in the absence and presence of anti-ST2. We also examined, in a separate study population, serum levels of soluble ST2 (sST2), before and after exposure in a pig barn and a bronchial LPS challenge. Farmers had signs of ongoing chronic inflammation with increased number of blood monocytes, and decreased expression of CD62L and CD162 on blood neutrophils. Farmers also had lower levels of sTLR2 and sCD14 in sputum and reduced expression of CD14 on sputum neutrophils than controls. Exposure to organic dust and LPS induced increase of serum sST2 in controls but not in farmers. In conclusion, farmers have signs of local and systemic inflammation associated with altered innate immunity characteristics.

## Introduction

A few hours of acute exposure in a pig barn induces acute airway inflammation with systemic involvement in healthy subjects [1]–[3]. In farmers who work in pig barns, inflammatory and physiologic responses to acute exposure are attenuated compared to naïve subjects [4]–[6] indicating some kind of tolerance, most likely caused by the daily exposure to organic dust. Thus, recruitment of inflammatory cells and cytokine production as well as increased bronchial responsiveness induced by exposure in pig barns is attenuated in farmers [4]–[6]. Farmers also have signs of an ongoing airway inflammation and higher prevalence of chronic inflammatory airway disorders, e g chronic bronchitis, than the population in general [7]–[9].

Certain farming environments are highly contaminated with airborne inhalable organic dust and concentrations up to 25 mg dust per m^3^ of air have been found in pig barns [10]. The dust contains various kinds of pathogen-associated molecular patterns (PAMPs) from fungi, Gram-positive and Gram-negative bacteria [11] that bind and activate a range of cell types trough toll-like receptors (TLRs). Binding of PAMPs to TLRs constitutes an important component of the innate immunity defence against invading microbes. The interaction leads to cell activation and expression of pro-inflammatory cytokines and type I interferons (IFNs) through activation of NF-κB and interferon regulatory factors (IRFs) [3]. Toll-like receptor 2 (TLR2) binds PAMPs such as peptidoglycans (PGN) from Gram-positive bacteria and TLR4 binds lipopolysaccharide (LPS) from endotoxin of Gram-negative bacteria, both abundant in pig barn dust [11], [12]. For the biological response, TLR4 is dependent on interaction with CD14, lipopolysaccaride binding protein (LBP) and the adapter molecule (MD-2). The TLRs need, however, strong negative regulatory mechanisms as too strong immune cell response may be harmful to the host [13], [14].

Tolerance to LPS is defined as reduced immune response to a repeated LPS challenge [15]–[17] and several of the intracellular negative TLR regulating systems, e g IL-1R-associated kinase (IRAK-M) and A20 are involved in tolerance to PAMPs. Suppression of tumorigenicity 2 (ST2L) is a transmembrane protein known to regulate TLR signaling [18]. The ST2L receptors are member of the interleukin (IL)-1R/TLR superfamily and share structural similarities with the TLRs by having the intracellular toll-interleukin 1 receptor (TIR) domain [19], [20]. ST2 have an essential role in development of tolerance to LPS [18], [21] and is involved in attenuation of TLR2 signalling [22]. Basophils and eosinophils express ST2 [23] constitutively and monocytes and Th2 cells express ST2 upon activation [15], [24]. The ligand of ST2 has recently been identified as IL-33, a member of the IL-1 superfamily [25]. Binding of IL-33 to ST2L on Th2 cells induces release of IL-4 and IL-13 by activation of NF-κB and MAP kinases [25]. Alternate splicing of ST2L generates a soluble variant of ST2 (sST2) [19] which increase in inflammatory disorders [26], [27]. Both ST2L and sST2 have negative regulating effects on TLR signalling [28].

An important biologic consequence of TLR signalling is production of chemoattractants leading to recruitment of inflammatory cells to the site of exposure. Adhesion proteins, expressed on inflammatory and vascular cells are of importance for cell migration from the circulation to the site of inflammation [29]. Farmers have increased serum levels of soluble L-selectin (sCD62L) and increased expression of CD11b [30], [31] on blood neutrophils which might be of importance for the attenuated exposure-induced cell recruitment observed in pig farmers [4], [31].

To further elucidate the altered innate immune responses in farmers one aim was to investigate whether the surface expression and soluble variants of pattern recognition receptors. Pig barn environment is known to contain high levels of gram^+^ and gram^−^ bacterial components and both pigs and pig farmers are frequently colonized with bacteria, such as methicillin resistant *Staphylococcus aureus* [32]. We aimed to focus on the surface expression of TLR2, TLR4 and its co-receptor CD14 on blood and sputum neutrophils and to measure concentration of the soluble variant of these receptors in serum and sputum. Another aim was to investigate if surface expression of proteins involved in cell migration (i.e. adhesion molecules, CD11b, CD62L and CD162) on blood and airway neutrophils (sputum) are altered in farmers compared with healthy previously unexposed controls. A further aim was to investigate the role of ST2 as a TLR regulator in farmers and controls. Therefore, sST2 was measured in serum after *in vivo* exposure in a pig barn and a bronchial LPS challenge, and it was explored whether blocking of the ST2 receptor influences the release of pro-inflammatory cytokines from peripheral blood cells stimulated with TLR ligands *ex vivo*.

## Materials and Methods

### Subjects and study design

In 15 pig farmers (2 females, mean age 45 (range 22–65) years) and 15 non-farming controls (2 females, mean age 51 (range 26–64) years) venous blood samples and induced sputum were collected.

For analysis of soluble ST2 in serum blood samples were collected in another 11 farmers, (1 female, mean age 41 (range 22–61) years) and 12 controls (2 females, mean age 33 (range 25–54) years) 1–2 weeks before and 7 hours after exposure in a pig barn and a bronchial LPS-challenge. Subject characteristics of these groups have been presented elsewhere [5], [6].

All subjects in the two groups of farmers and non-farming controls, were non-smokers and had no history of COPD, asthma or atopic disease, the latter verified by a negative skin prick test to 17 common allergens. All had normal lung function assessed by spirometry performed according to the ATS recommendations [33] (table 1). The study was approved by the Karolinska Institute Ethics Committee (Stockholm, Sweden), and all individuals gave their informed written consent to participate.

**Table 1:** Baseline subject characteristics.

	*Controls (n = 15)*	*Farmers (n = 15)*
*Duration of work in pig barn* *Mean (range) Years*	-	16(2–35)
*Work in pig barns* *Mean (range) hours/day*	-	3.5(0.5–8.0)
*Number of pigs in the barn* *Mean (range)*	-	2066(60–6000)
*FEV_1_ L mean* *(95% CI)*	4.02(3.69–4.36)	3.93(3.38–4.47)
*FEV_1_ % predicted mean* *(95% CI)*	100(96–105)	94(85–103)
*VC L mean* *(95% CI)*	5.08(4.70–5.47)	4.87(4.21–5.54)
*VC % predicted mean* *(95% CI)*	95(91–100)	89(82–97)
*FEV_1_/VC % mean* *(95% CI)*	79(76–81)	80(77–83)

Baseline subject characteristics CI: Confidence interval; FEV1: forced expiratory volume in 1 s; VC: vital capacity. Between-group comparisons (lung function) were assessed by ANOVA and Fisher's protected lest significant difference (PLSD). * indicates p<0.05 compared with controls.

### Pig barn and LPS exposure

The exposure in pig house and the LPS-challenge were performed as previously described [5], [6]. The subjects underwent 3 hours exposure in a pig barn while weighing pigs and the challenge with LPS (53.4 µg) was performed by inhaling LPS solution (*Escherichia coli* serotype 0111:B4, 1.25 mg/ml (SIGMA)) with an inhalation dosimeter (SPIRA® Elektro 2, Hameenlina, Finland). There was a minimum of three weeks between pig house exposure and LPS challenge which were performed in random order.

### Peripheral blood sampling

Peripheral venous blood was collected in ethylene diamine-tetra-acetic acid (EDTA) vacutainer tubes (BD Bioscience, San Jose, California) for assessing cell surface markers and in heparinized tubes (BD Bioscience) for *ex vivo* stimulations.

### Cell distribution in peripheral blood

To determine the absolute cell number in peripheral blood, TruCOUNT™ tubes containing a specified number of beads were used. Whole blood and a four–colour antibody mixture (CD3FITC/CD8PE/CD45PerCp/CD4APC from BD Bioscience) were added to a TruCOUNT tube (BD Bioscience) and incubated in the dark at 20–22°C for 15 min. To lyse red blood cells, 450 µl BD PharM Lyse™ (BD Bioscience Pharmingen, San Jose, Carlifornia) was added and an additional 10 min incubation, in the dark at 20–22°C, was performed. All samples were then analysed on a FACSCalibur™ (BD Bioscience) using MultiSet™ (BD Bioscience) to perform a five-part white blood cell differential.

### Sputum induction, processing and cell count

Sputum induction was performed as previously described [34]. Following salbutamol inhalation, saline was inhaled in increasing concentrations (3, 4, 5%) and the participants were asked to cough deeply and make an attempt to expectorate sputum. The saliva-free sample was considered sufficient at a weight ≥1000 mg. After filtration the sputum sample was centrifuged for 10 minutes at 400 *g* and cell count and viability test (trypan blue) were performed. Slides were also prepared by cytocentrifugation and stained with May-Grünwald Giemsa and 300 cells were assessed for differential cell counts. Samples with less than 100 cells were not accepted for an accurate differential count. All sputum samples contained less than 40% squamous cells and were therefore included in the analyses.

### Surface markers on peripheral blood and sputum neutrophils

**Figure 1:**
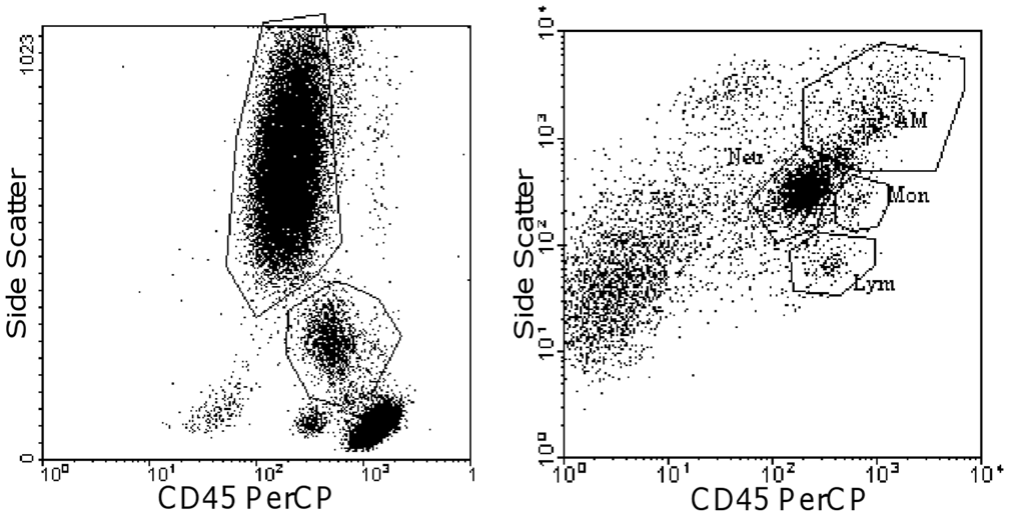
Gating strategies for peripheral blood and sputum neutrophils. Gating strategies for blood neutrophils, side scatter versus FL-3 (CD45 PerCp) (A), and sputum neutrophils, side scatter (logarithmic scale) versus FL-3 (CD45 PerCp) (B).

To evaluate the expression of TLRs, CD14 and adhesion molecules on peripheral blood neutrophils, whole blood was stained with fluorochrome-labeled monoclonal antibodies. Blood was incubated for 20 minutes in the dark at 20–22°C with titrated amounts of anti-CD45-PerCp together with anti-TL2-PE (eBioscience), anti-TLR4-PE (eBioscience), anti-CD11b-PE (BD Bioscience), anti-CD14-FITC (eBioscience), anti-CD62L-PE (BD Bioscience) or anti-CD162-PE (BD Bioscience). Red blood cells were lysed by using PharM Lyse™ (BD Bioscience Pharmingen) and then washed twice. Sputum cells (10^5^) were stained for 30 minutes in the dark at 4°C with titrated amounts of the same monoclonal antibodies as peripheral blood. As negative controls, isotype matched antibodies were used. Samples were analysed using FACSCalibur™ (BD Bioscience) flow cytometry and CELLQuest™ software. To gate on blood and sputum neutrophils, forward scatter, side scatter and FL-3 (CD45-PerCp) were used (figure 1). Results are presented as relative median fluorescence intensity; rMFI (rMFI = monoclonal antibody/matched isotype control) to obtain objective results.

### Soluble ST2 (sST2), sCD14, IL-6 and CXCL8 in serum and sputum

Soluble ST2 and sCD14 were measured in serum and sputum by using DuoSet ELISA kits (R&D systems, Europe, Abingdon, UK). The detection range for sST2 and sCD14 was 15.6–2000 pg/ml and 60–4000 pg/ml, respectively. As IL-6 levels in sputum were anticipated to be low a sensitive in-house ELISA [35], (more sensitive than CBA, see below) was used. The same method was used for CXCL8 using commercially available antibody pairs (R&D systems, Europe, Abingdon, UK). The detection range for IL-6 and CXCL8 were 2.8–375 pg/ml and 40–3200 pg/ml, respectively. For duplicate samples an intra-assay coefficient (CV)<10% was accepted for serum and <15% was accepted for sputum.

### *Ex vivo* stimulation of peripheral blood

Heparinized peripheral blood (250 µl) was incubated in an equal volume of RPMI 1640 containing 2 mmol/l glutamine (Sigma–Aldrich) for 4 h in 37°C. During the incubation, blood was stimulated with media alone, the TLR4 ligand LPS (*Escherichia coli* serotype 0111:B4, Sigma–Aldrich; 10 ng/ml and 1000 ng/ml), the synthetic TLR2 ligand Pam3Cys (EMC microcollections GmbH, Tübingen, Germany; 10 ng/ml and 1000 ng/ml), TNF (R&D systems, Europe, Abingdon, UK, 10 ng/ml), organic dust from a pig barn (collected in the pig barn on shelves and window ledges about 1.2 m above the floor; 10 ng/ml and 1000 ng/ml), IL-33 (R&D systems; 1 ng/ml and 10 ng/ml), and IL-33 (1 ng/ml) together with LPS (10 ng/ml and 1000 ng/ml). Anti-ST2 (R&D systems; 5 µg/ml) and matched isotype as negative control (R&D systems) were used in 5 farmers and 5 controls, randomly chosen from the study group. The blood was first incubated for 1 h with the antibodies only and the following 4 h in the presence of media, LPS, , in above mentioned concentrations. Individual data are presented as mean of two observations.

### Cytometric bead array (CBA)

Based on previous studies, we anticipated the cytokine levels to be fairly high, and therefore cytometric bead array (BD™ CBA, BD Bioscience) was used to measure IL-6 and TNF after *ex vivo* stimulations. Samples were first diluted if needed in BD™ CBA Assay Diluent and then incubated with anti-IL-6 and anti-TNF coated beads for 1 hour at 20–22°C. Phycoerythrin (PE) conjugated IL-6 and TNF detection antibodies were added and followed by 2 hours incubation in dark at 20–22°C. After incubation, samples were washed and diluted in BD™ CBA Wash Buffer. Samples were analysed using FACSCalibur™ (BD Bioscience) flow cytometry and CELLQuest™ Software and FCAP Array™ Software. The results are presented as pg/ml. The standard curve was supplemented with extra standard points giving a detection range between 5–5000 pg/ml for both IL-6 and TNF.

### Statistics

Data are presented as scattergrams or median and 25^th^–75^th^ percentiles unless otherwise stated. Statistical analyses were performed using Friedmans test followed by Wilcoxon Signed Rank when appropriate (within group comparisons) or Mann-Whitney U test (between group comparisons). Lung function data were considered to be normally distributed and analysed by means of ANOVA and Fisher's protected least significant difference (PLSD). A P-value<0.05 was considered significant.

## Results

Due to low cell yield and technical problems connected with sampling of the material some data are missing. Number of subjects is 15 each group unless otherwise stated.

### Cell distribution in peripheral blood and sputum

The concentration of monocytes in peripheral blood was higher in farmers than in controls (p = 0.024) whereas other blood cell types did not differ between the groups (table 2).

**Table 2:** Concentration of leukocyte subsets in peripheral blood.

	*Controls cells×10∧9/L blood*	*Farmers cells×10∧9/L blood*	*P-value*
*Neutrophils*	2.99 (2.40;4.11)	3.57 (3.21;4.44)	0.221
*Monocytes*	0.44 (0.41;0.51)	0.55 (0.49;0.57)	0.024
*Lymphocytes*	2.07 (1.74;2.15)	2.17 (2.00;2.40)	0.093
*Basophils*	0.05 (0.04;0.07)	0.06 (0.05;0.07)	0.254
*Eosinophils*	0.15 (0.65;0.20)	0.11 (0.09;0.19)	0.678

Concentration (medians and 25^th^–75^th^ percentiles) of neutrophils, monocytes, lymphocytes, basophils, and eosinophils in peripheral blood. Results are present as cells×10^9^/L blood (controls n = 15 and farmers n = 15). P-values indicate differences between the groups.

The macrophage number in sputum was lower in farmers than in the controls (p<0.001), otherwise cells in sputum did not differ between the groups (data not shown).

### Surface markers on peripheral blood and sputum neutrophils

**Figure 2:**
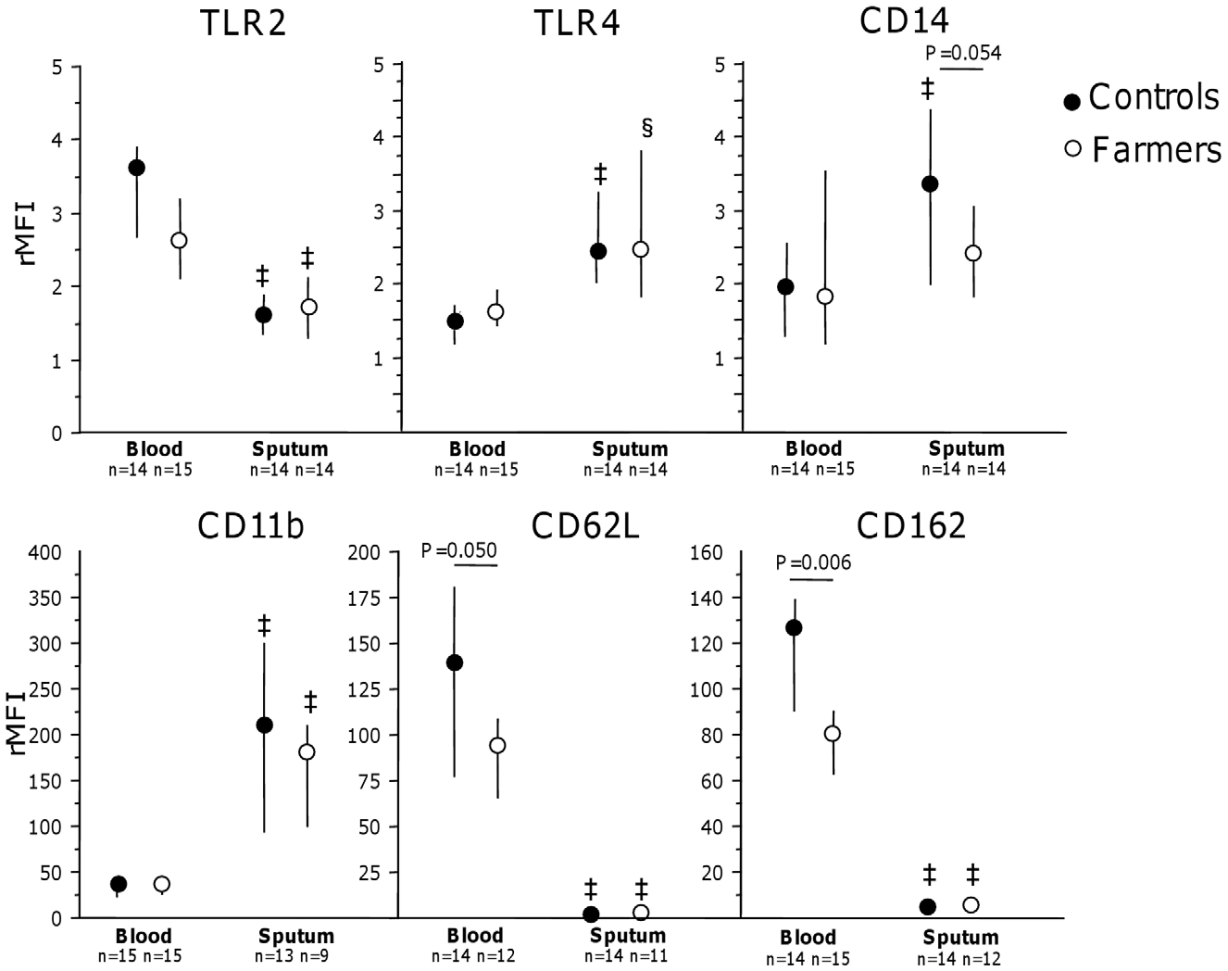
Surface expression of pattern recognition receptors and adhesion proteins on blood and sputum neutrophils. Surface expression of TLR2, TLR4, CD14, CD11b, CD62L and CD162 on blood and sputum neutrophils. P-values indicate differences between the groups. rMFI = relative mean fluorescence intensity. Medians and 25^th^–75^th^ percentiles are presented. § indicates p<0.05 and ‡ indicates p<0.01 for within group comparisons between blood and sputum.

The expression of TLR2, TLR4 and CD14 on peripheral blood and sputum neutrophils did not differ between farmers and controls although CD14 expression tended to be lower in farmers (p = 0.054; figure 2). The expression of TLR2 was lower and TLR4 was higher on sputum than on blood neutrophils in farmers (p = 0.008 and p = 0.013, respectively) and controls (p = 0.005 and p = 0.004, respectively, figure 2). The expression of CD14 was higher on sputum than on blood neutrophils in controls (p = 0.006) but not in farmers (p = 0.033 between the groups; figure 2).

The expression of CD62L and CD162 on peripheral blood neutrophils was lower in farmers than in controls (p = 0.050 and p = 0.006, respectively), whereas the expression of CD11b on peripheral blood neutrophils and CD11b, CD62L and CD162 on sputum neutrophils did not differ between the groups (figure 2).

The expression of adhesion molecules CD11b was enhanced and CD62L and CD162 were decreased on sputum neutrophils compared with neutrophils in blood in both groups (p≤0.008; figure 2).

### Serum and sputum supernatant analyses

**Figure 3:**
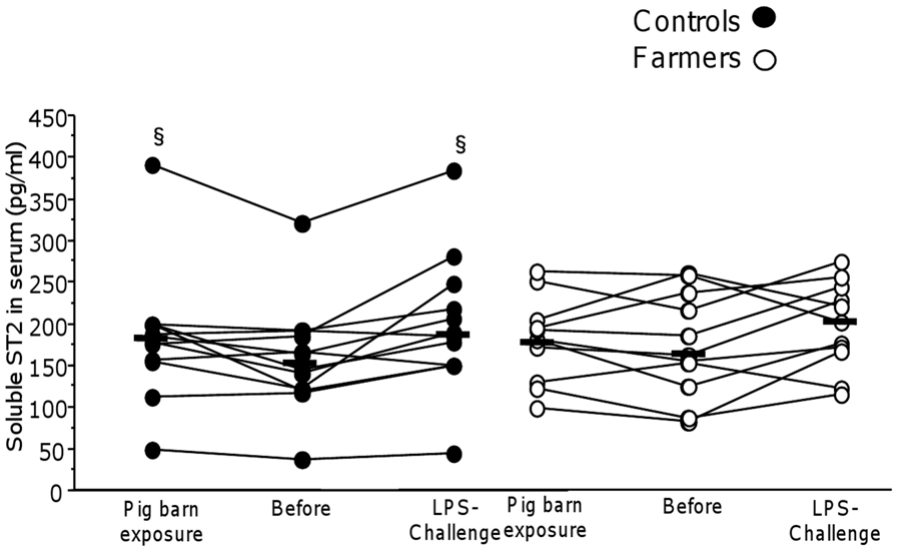
Concentration of sST2 in serum before and after exposure in a pig barn and a LPS challenge. Concentration of sST2 in serum before and after exposure in a pig barn and a LPS challenge in 11 farmers and 12 healthy non-exposed control subjects. Horizontal lines indicate median values. § indicate p<0.05 for pre- and post-exposure comparisons.

Serum levels of sST2 did not differ between the groups (figure 3) and the levels of sST2 in sputum were below detection limit in all subjects (data not shown).

Serum levels of ST2 increased following pig house exposure (p = 0.034) and LPS challenge (p = 0.008) in the controls only, with no differences between the groups (figure 3).

**Figure 4:**
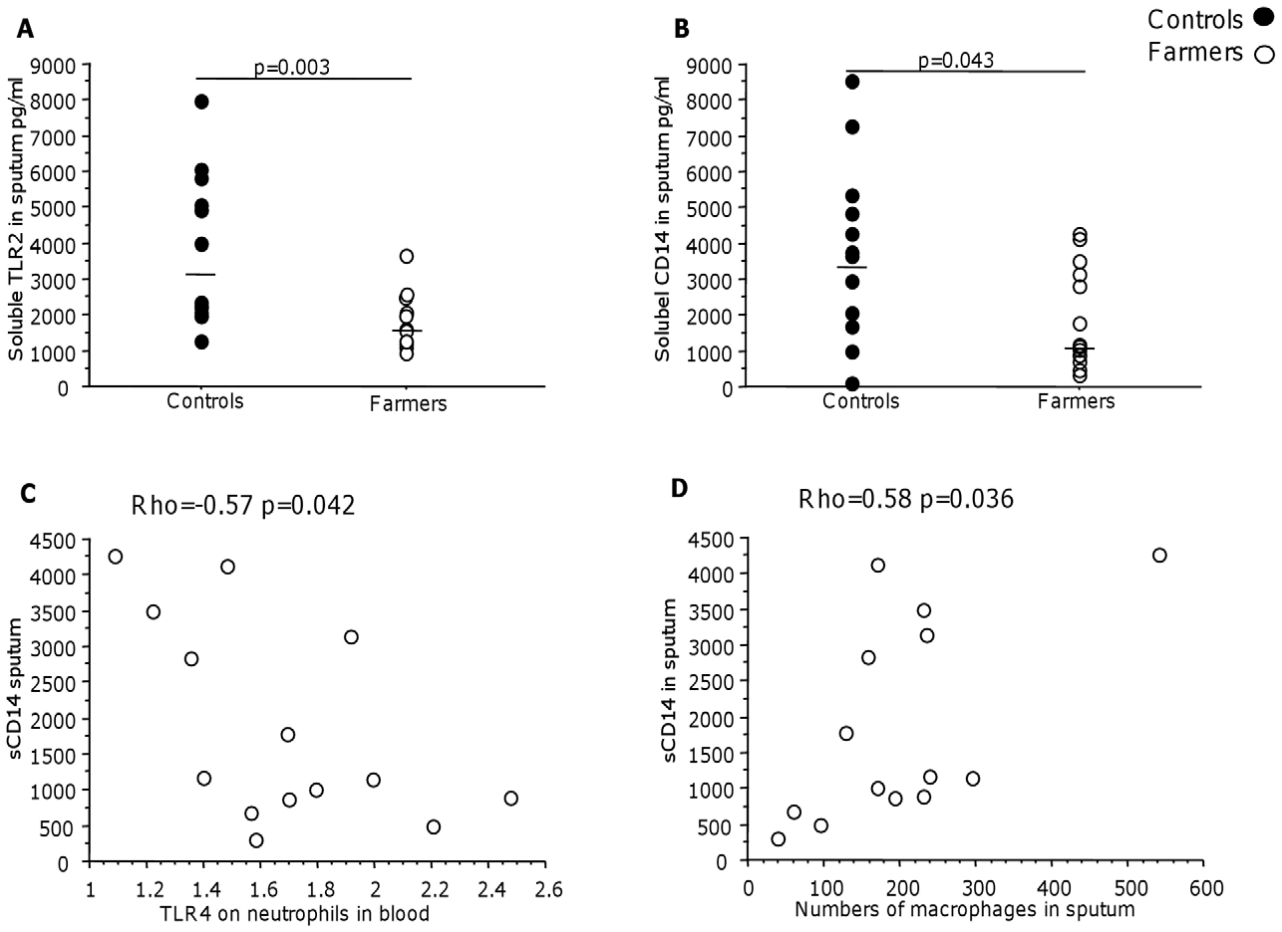
Concentration of sTLR2 and sCD14 in sputum and correlation between sputum sCD14 and TLR4 expression on blood neutrophils. Concentration of sTLR2 (A) and sCD14 (B) in sputum (controls n = 14 and farmers n = 14). P-values indicate differences between the farmers and controls. A negative correlation between sputum sCD14 and TLR4 expression on blood neutrophils (C), and a positive correlation between sCD14 and macrophages number in sputum in farmers (D).

Soluble TLR2 (sTLR2) and sCD14 in sputum were lower in farmers than in controls (figure 4) but sCD14 in serum did not differ between the groups (data not shown). In farmers there was a negative correlation between sCD14 in sputum and TLR4 expression on blood neutrophils and a positive correlation between sCD14 and macrophage number in sputum (figure 4).

There was no difference in IL-6 and CXCL8 levels in sputum between the groups (data not shown).

### *Ex vivo* stimulation of peripheral blood

**Figure 5:**
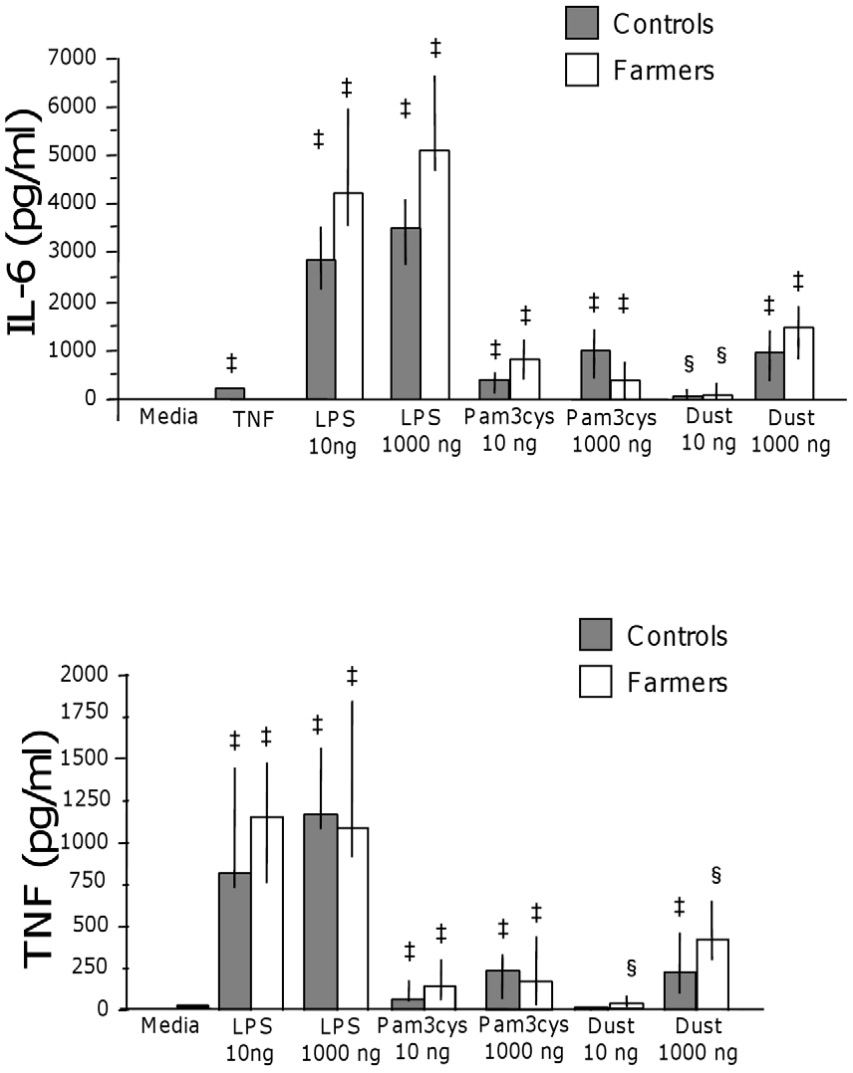
Release of IL-6 and TNF after *ex vivo* stimulation of peripheral blood. Release of IL-6 and TNF after *ex vivo* stimulation of peripheral blood with TNF, LPS, Pam3Cys or pig barn dust (controls n = 13 and farmers n = 14). § indicates p<0.05 and ‡ indicates p<0.01 compared with medium control.

*Ex vivo* stimulation of peripheral blood with LPS and Pam3cys increased IL-6 and TNF in farmers and controls (p≤0.002 for all; figure 5).

Stimulation of peripheral blood with pig barn dust (10 ng/ml) induced an increase of IL-6 in farmers (p = 0.028) and controls (p = 0.011) and an increase of TNF only in the farmers (p = 0.028). At the highest dust concentration there was an increase of IL-6 and TNF in both groups (p≤0.002; figure 5). Tumour necrosis factor (10 ng/ml) induced IL-6 increase only in the controls (p = 0.002) (figure 5). There were no differences in IL-6 and TNF increase between the farmers and controls following LPS, Pam3cys and dust stimulation.

**Figure 6:**
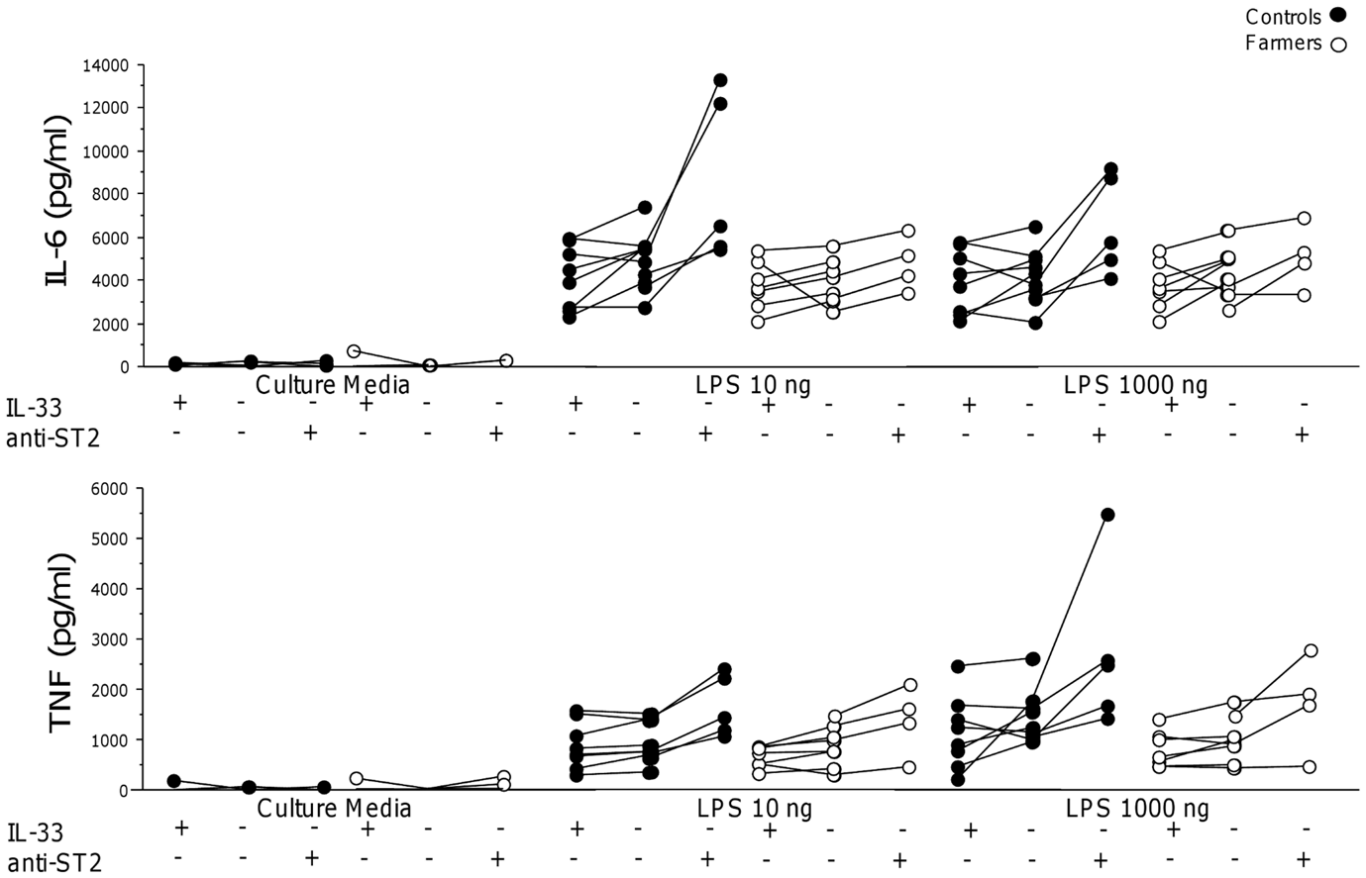
Release of IL-6 and TNF after *ex vivo* stimulations of peripheral blood in the presence or absence of anti-ST2. Release of IL-6 and TNF after ex vivo stimulations of peripheral blood presence or absence of anti-ST2 with LPS in presence or absence of anti-ST2 (controls n = 5 and farmers n = 4) or IL-33 (controls n = 8 and farmers n = 7).

Anti-ST2 antibodies induced a further increase of IL-6 and TNF release beyond the effect of LPS stimulation in controls (figure 6). Release of IL-6 and TNF was not influenced by IL-33 in LPS stimulated blood in neither group (figure 6). Analyses of pooled data from both groups revealed that IL-33 induced a decrease in TNF release in LPS stimulated blood (p = 0.027 at LPS 10 ng) and a slight decrease in IL-6 (p = 0.061 at LPS 1000 ng; data not shown).

## Discussion

The present study clearly demonstrates that continuous exposure to organic material alters innate immune responses. Thus, daily exposure to high levels of pathogen-associated molecular patterns (PAMPs) was associated with lower number of macrophages and lower levels of soluble CD14 (sCD14) and TLR2 (sTLR2) in sputum from farmers compared with unexposed healthy subjects. The expression of CD14 on sputum neutrophils and adhesion molecules (CD62L and CD162) on peripheral blood neutrophils was lower in farmers than in controls and exposure to organic dust and LPS increased sST2 levels in serum in the control subjects but not in the farmers. The increased concentration of blood monocytes and decreased expression of CD62L (L-selectin) and CD162 (P-selectin) on blood neutrophils in farmers suggest a systemic engagement. This is supported by previous findings of reduced surface expression of CD62L and CD162 on neutrophils following activation with TNF and leukotriene B_4_ [36]. Reduced surface expression of CD62L on blood neutrophils has been found in other conditions characterized by systemic inflammation, such as type- 2-diabetes with micro-angiophaty [37]. It has been shown that pig farmers have elevated serum levels of soluble L-selectin, probably due to increased shedding of membrane bound protein [31]. Our findings are intriguing considering that recruitment of neutrophils into the blood and airways following acute exposure in a pig barn and into the airways following LPS-challenge are attenuated in pig farmers compared with controls [38]. The causes for the impaired neutrophil migration in farmers may be a consequence of impaired release of CXCL8 [4], [38] and reduced expression of adhesion molecules on blood neutrophils. The expression of CD62L and CD162 was reduced and CD11b expression was enhanced on sputum neutrophils compared with peripheral blood neutrophils which are most likely due to activation of neutrophils during the migration process during transition from blood into the airways. This activation seems to be independent of previous or ongoing exposure as we found a similar response in the regularly exposed farmers and naïve controls.

It has been suggested that sTLR2 has an important role as a negative or first line regulator of membrane bound TLR2 by binding TLR2 ligands, thereby limiting the activation of membrane bound TLR2 in order to reduce harmful host effects [39], [40]. Thus, adding recombinant sTLR2 to monocytic culture strongly attenuated cell responses to TLR2 stimuli [40]. The reduced levels of sTLR2 in sputum in farmers are likely related to inhalation of high levels of TLR2 ligands which directly bind to produced sTLR2. Monocytes release sTLR upon activation [39] and it could be anticipated that monocytes still release sTLR2 after transformation into macrophages which in turn indicates a causal relationship between the decreased numbers of macrophages in sputum the lower levels of sTLR2 in the farmers. It has not been clearly shown that neutrophils, like monocytes [39], release sTLR2 upon activation. However, it could be speculated that also neutrophils release TLR2 which may explain the low expression on sputum neutrophils.

Chronic exposure in pig barn environment seems to affect the CD14 expression as the expression on sputum neutrophils was lower in farmers than in controls. Farmers have signs on neutrophilic airway inflammation [41], [42] and it could be speculated that the reduced CD14 expression on sputum neutrophils from farmers is caused by increased levels of elastase known to be increased in neutrophilic inflammation. Neutrophil elastase has a proteolytic effect on membrane bound CD14 by releasing the receptor into extracellular medium [43]. As farmers have increased proportion of blood Th2 cells producing IL-4 and IL-13 [5], this may contribute to the explanation as Th2 cytokines, e g IL-4, have the ability to reduce surface expression of CD14 [44], [45].

We observed decreased sputum levels of sCD14 in the farmers. Farmers regularly inhale high amounts of LPS that binds to sCD14 which therefore may not be detectable with the ELISA method used. It might also be related to the decreased proportion of sputum macrophages in farmers as we observed a correlation between sCD14 and number of sputum macrophages indicating that macrophages may be an important producer of sCD14.

Increased levels of sST2 are induced by pro-inflammatory stimuli such as LPS and TNF [46]. Serum levels of sST2 are increased in several inflammatory disorders [26], [47]–[49] and intravenous injection of LPS increases serum levels of sST2 dramatically in healthy subjects [15]. The attenuated response in serum sST2 release after exposure in a pig house and LPS challenge in the farmers might be due to tolerance. As systemic cytokine responses are attenuated in farmers [4] this may, in part, explain the weaker sST2 increase in serum following exposure in pig barns in this group.

We have, in a previous study, observed increased concentration of blood neutrophils in farmers compared to unexposed controls, a finding that was not confirmed in the present study. This might be due to differences in exposure between the participating farmers. There is heterogeneity in previous exposure within the group of pig farmers with great variation in the cumulative exposure depending on the duration of employment and length of the working days. Thus, the possible difference in pre-trial cumulative exposure and the small study groups (15 pig farmers and 15 controls) may have influenced the possibilities to detect differences between the groups. However, despite this our study revealed some relevant and important new findings.

In conclusion, farmers showed altered expression of adhesion proteins on blood neutrophils, altered expression and soluble levels of PRR in the airways and attenuated release of sST2 after exposure in a pig barn and after a bronchial LPS challenge. The altered response in the farmers is probably caused by chronic exposure in pig barn environment and may be of pathogenic importance in development of chronic airway diseases, such as chronic bronchitis and increased occurrence of colonization of bacteria in the airways, conditions that are frequently observed in this group [32].
